# Quantitative Evaluation of Contamination on Dental Zirconia Ceramic by Silicone Disclosing Agents after Different Cleaning Procedures

**DOI:** 10.3390/ma8052650

**Published:** 2015-05-15

**Authors:** Sebastian Wille, Vincent van Broeck, Thomas Strunskus, Franz Faupel, Matthias Kern

**Affiliations:** 1 Department of Prosthodontics, Propaedeutics and Dental Materials, School of Dentistry, Christian-Albrechts University, Arnold-Heller-Straße 16, Kiel D-24105, Germany; E-Mail: mkern@proth.uni-kiel.de; 2 Chair for Multicomponent Materials, Institute for Materials Science, Faculty of Engineering, Christian-Albrechts University at Kiel, Kaiserstr. 2, Kiel D-24143, Germany; E-Mails: vivb@tf.uni-kiel.de (V.B.); ts@tf.uni-kiel.de (T.S.); ff@tf.uni-kiel.de (F.F.)

**Keywords:** contamination, zirconia, silicone, cleaning, surface analysis

## Abstract

The aim of this study was to evaluate the effectiveness of cleaning procedures for air-abraded zirconia after contamination with two silicone disclosing agents. Air-abraded zirconia ceramic specimens (IPS e.max ZirCAD) were contaminated with either GC Fit Checker white or GC Fit Checker II. Untreated zirconia specimens were used as control. Afterwards the surfaces were cleaned either with waterspray or ultrasonically in 99% isopropanol or using a newly developed cleaning paste (Ivoclean). After cleaning X-ray photoelectron spectroscopy (XPS) was performed and the relative peak intensities of Zr, C and Si were used for a qualitative comparison of the residuals. There was no significant difference between the two different silicone disclosing agents. An additional cleaning step with isopropanol led to a significantly lower amount of residuals on the surface, but an additional cleaning process with Ivoclean did not reduce the amount of carbon residuals in comparison to the isopropanol cleaning. Just the silicone amount on the surface was reduced. None of the investigated cleaning processes removed all residuals from the contaminated surface. Standard cleaning processes do not remove all residuals of the silicone disclosing agent from the surface. This may lead to a failure of the resin-ceramic bonding.

## 1. Introduction

In the last decade, zirconia restorations have become more and more important in dental applications. Due to its mechanical properties and also for esthetic reasons zirconia ceramics are widely used as fixed partial prostheses (FDPs), resin-bonded FDPs, full-coverage crowns, implant abutments, and endodontic posts [1]. In comparison to conventional cementation, which in most cases can also be used for luting zirconia restorations, durable resin-ceramic bonding is required for restorations with little or no mechanical retention such as resin-bonded retainers for fixed dental prostheses [2,3].

It has been shown in several *in vitro* studies that a strong, durable resin-zirconia bonding can be achieved by using a 10-methacryloxydecyl dihydrogenphosphate (MDP)-containing composite resin after air-abrasion with alumina particles [4,5,6,7,8,9]. These studies have been performed under well-controlled and clean conditions in the laboratory. However, in clinical situations zirconia ceramic bonding surfaces are contaminated by saliva, blood or silicone indicators during try-in of the restoration. As the set silicone shows a high silicone stability it might be assumed that no residuals are left on the surface after removal of the silicone disclosing agents. However, Yang *et al.* showed by XPS investigations that the surface is contaminated with silicone residuals after the use of silicone disclosing agent (Fit checker, GC) even after cleaning procedures including water rinsing or isopropanol rinsing [10]. In the same study the authors showed that contaminations on the zirconia surface lead to a dramatic drop in tensile bond strength. The reason for the contamination has been explained in several ways. Some older publications explain it by a chemical reaction between the silicone disclosing agent and the restoration surface leading to a stable contamination on the ceramic surface [11,12]. Some investigators also assumed that a not polymerized thin film is left on the surface that leads to a reduction in the bond strength [12,13].

However, meanwhile the company GC developed another product, the so-called Fit Checker II which is also on the market. The different compositions of both products are given in Table 1. Although both base materials are mainly using polymers from the group of the silicones, there are large differences in the compositions. The base material of Fit Checker white consists also of calcium carbonate whereas Fit Checker II contains silicon dioxide. The catalyst materials are completely different in their compositions. In the product description of Fit Checker II it is claimed that there will be a clean, residue free surface after peeling it away.

The aim of this study was to compare the older material Fit Checker white with Fit Checker II concerning the cleanability on zirconia surfaces. In this study cleaning procedures were chosen that are generally clinically available namely water spray and ultrasonic cleaning in isopropanol. In addition, also a newly developed cleaning medium (Ivoclean, Ivoclar Vivadent) was tested. Ivoclean consists of an alkaline suspension of zirconium oxide particles. As claimed by its manufacturer to the size and concentration of the particles in the medium, contaminants are much more likely to bond to them than to the surface of the ceramic restoration. Therefore, this cleaning medium is supposed to absorb contaminants and thus leaving behind a clean zirconia surface. Another aim of the study was to check, whether the easy cleaning procedures that are generally available are really efficient to remove contaminations in a way that the bond strength will not be significantly reduced.

**Table 1:** Used materials, their composition as given by the manufacturers in wt-% and their batch numbers.

Material	Company	Composition	Batch No.
IPS e.max ZirCad	Ivoclar Vivadent	ZrO_2_: 87%–95%, Y_2_O_3_: 4%–6%, HfO_2_: 1%–5%, Al_2_O_3_: 0%–0.5%	L48411
GC Fit Checker white—Base	GC Corporation	Dimethyl methylhydrogenpolysiloxane: 65% CaCO_3_: 35%	1302221
GC Fit Checker white—Catalyst	GC Corporation	Petrolatum: 55%, Ethylsilicate: 40%, fatty acid salt: 5%	1302221
GC Fit Checker II Base	GC Corporation	SiO_2_: 40%–60%, Polydimethyl siloxane: 38%–42%	1305151
GC Fit Checker II Catalyst	GC Corporation	SiO_2_: 42%–45%, Vinyldimethylpolysiloxane: 50%–60%	1305151
Ivoclean	Ivoclar Vivadent	ZrO_2_: 10%–15%, H_2_O: 65%–80%, PEG: 8%–10%, NaOH: <1%, Pigments, Additives: 4%–5%	P66483

## 2. Results and Discussion

### 2.1. Results

**Figure 1:**
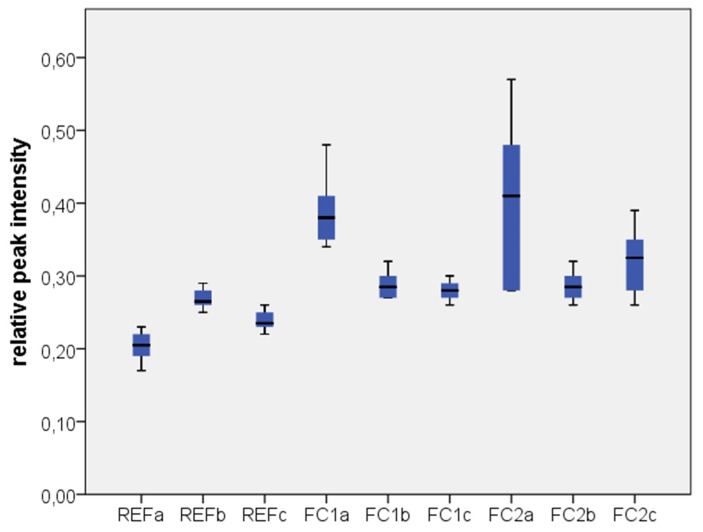
Relative peak intensity of carbon (group codes see Table 2).

**Figure 2:**
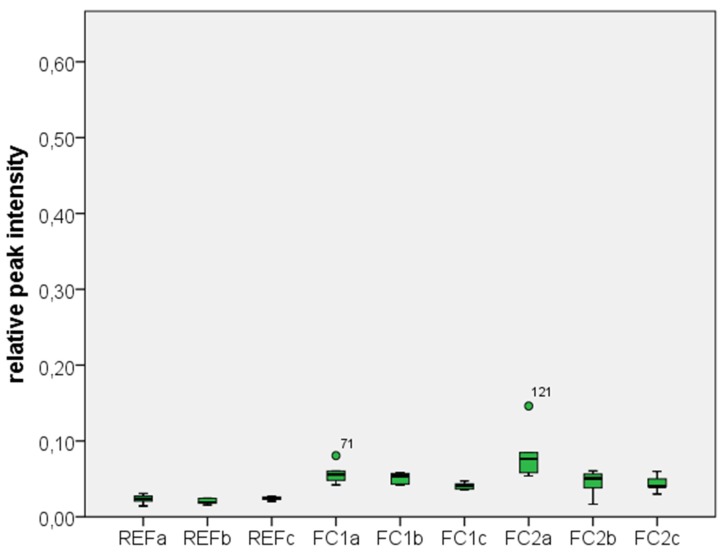
Relative peak intensity of silicone (group codes see Table 2).

The relative line intensities (areas) of the X-ray photoelectron spectroscopy (XPS) measurements of the C 1s, Si 2p and Zr 3d lines and the corresponding standard deviations for the different groups are shown in Table 2. There was no significant difference between the main groups FC1 and FC2 (*p* = 0.350). The two-way ANOVA with a post-hoc Tukey’s HSD test also revealed that the cleaning process with an additional step of an ultrasonic bath in isopropanol showed a significant decrease in the relative peak intensities of C (*p* = 0.006) and Si (*p* = 0.005) for the previously contaminated specimens, whereas the additional cleaning with Ivoclean led only to a minor decrease of the Si amount on the surface as shown in Figure 1 and Figure 2. However, this decrease was statistically not significant (*p* = 0.702). Independently of the conducted cleaning processes, all previously contaminated specimens showed a significantly higher amount of C and Si on the surface than the uncontaminated reference groups (*p* ≤ 0.001).

### 2.2. Discussion

The results of this study show that there are always some residuals of the silicone disclosing agent after the conducted cleaning processes left on the surfaces. As it has been shown by Yang *et al.*, these residuals can lead to a drastic decrease in the bond strength [14]. Also additional cleaning processes with Ivoclean and isopropanol do not remove all residuals from the silicone disclosing agents, although the amount of contamination is significantly decreased. In order to achieve a reliable bonding to zirconia ceramics one needs to use other cleaning processes that are more efficient, such as for example air abrasion with alumina particles [15]. However, this method might aggravate the degradation of zirconia [16].

Applying phosphoric acid etching did not lead to the removal of silicone residuals as Zhang *et al.* have shown on a nano-alumina coated zirconia surface [17]. Several studies concerning the use of plasma treatment on zirconia surfaces show also promising results that this method might be an efficient cleaning process for chair side application with less surface damaging effects [18,19].

**Table 2:** X-ray photoelectron spectroscopy (XPS) results: mean relative elemental intensities and corresponding standard deviations of Zr, C and Si of the test groups determined from the XPS line intensities (areas).

Test Group	Zr	C	Si
REF a	0.7723 ± 0.0207	0.2033 ± 0.0216	0.0234 ± 0.0057
REF b	0.7100 ± 0.0141	0.2683 ± 0.0147	0.0201 ± 0.0037
REF c	0.7350 ± 0.0152	0.2383 ± 0.0147	0.0243 ± 0.0024
FC1 a	0.5517 ± 0.0488	0.3900 ± 0.0522	0.0571 ± 0.0134
FC1 b	0.6633 ± 0.0163	0.2883 ± 0.0194	0.0514 ± 0.0071
FC1 c	0.6783 ± 0.0184	0.2800 ± 0.0141	0.0410 ± 0.0043
FC2 a	0.5117 ± 0.1277	0.4050 ± 0.1161	0.0826 ± 0.0334
FC2 b	0.6683 ± 0.0240	0.2867 ± 0.0216	0.0455 ± 0.0162
FC2 c	0.6367 ± 0.0513	0.3217 ± 0.0471	0.0411 ± 0.0099

Notes: REF: no contamination; FC1: contamination with Fit Checker white; FC2: contamination with Fit Checker II; a: water spray cleaning; b: additional ultrasonic bath with isopropanol; c: water spray, Ivoclean, water spray and ultrasonic bath with isopropanol.

No differences between the two silicone disclosing media GC Fit Checker white and GC Fit Checker II could be detected regarding the evaluated cleaning procedures. The XPS measurements show that the contamination of the surface with silicon and carbon based molecules can be reduced considerably by ultrasonic cleaning in 99% isopropanol in comparison to the simple water spray cleaning. However, the additional cleaning process with Ivoclean had only a small influence on the surface concentration of silicon. There was no significant change in the carbon concentration. Taking into account that Yang *et al.* have shown that carbon based residuals have a larger effect on the tensile bond strength than silicone residuals [14], the additional use of Ivoclean after the application of silicone disclosing agents might be questioned.

One limitation of this study is that the XPS measurements do not give any information about the structure of the contaminants on the surface. So it is unknown, whether the contaminations build a very thin film or single islands on the surface. The relatively large standard deviation in the first subgroup might be due to the fact that the water spraying was performed manually. This kind of cleaning was just a rough cleaning in order to remove visible contaminants. However small variations in the hardening and application process of the silicone disclosing agents might also have led to relatively large variations in this group. Another limitation of this study is that only silicone disclosing agents have been included. However, other clinical relevant contaminations such as e.g., saliva had not been considered.

## 3. Experimental Section

### 3.1. Specimen Preparation

Fifty-four pre-sintered IPS e.max ZirCAD specimens (Ivoclar Vivadent, Schaan, Liechtenstein) have been sectioned (10 mm × 10 mm × 1.5 mm) with a precision saw ISOMET 1000 (Buehler, Düsseldorf, Germany). Afterwards the zirconia specimens were ground with 600-grit silicon carbide paper before the final sintering process was performed according to manufacturer’s instructions. Then the specimens were air-abraded with 50 µm alumina particles at 0.1 MPa using a P-G 400 (Harmisch+Rieth, Cologne, Germany) and cleaned in an ultrasonic bath with 99% isopropanol (Otto Fischer, Saarbrücken, Germany) for 3 min. After drying of the specimens with oil-free air at 0.33 MPa the specimens were divided into three main groups.

The specimens of two groups (FC1 and FC2 respectively) where treated with two different silicone disclosing agents. Therefore the components of the flowing silicone disclosing agents, GC Fit Checker white or GC Fit Checker II (GC Corporation, Tokyo, Japan) respectively, were mixed and applied to the specimens within 30 s. After the silicone disclosing agent had been specimen to one specimen, another specimen was slightly pressed on top of the covered surface and both zirconia specimens with the silicone disclosing agent in between were stored for 3 min. Finally the specimens were separated manually and the hardened silicone disclosing agent was removed. The third group was not contaminated before the cleaning processes were performed and are used as reference group (REF).

### 3.2. Cleaning Procedures

The cleaning procedures of the main groups FC1 and FC2 were performed subsequently after the contamination process. The specimens of all three main groups were divided into three subgroups with different cleaning procedures. Every wet cleaning step with isopropanol or water spray was followed by a drying step with air as it is described previously. The zirconia ceramic specimens of the first subgroup were only cleaned with water spray with a pressure of 0.15 MPa for 20 s (a), for the second subgroup additionally a cleaning step in an ultrasonic bath with 99% isopropanol was conducted for 3 min (b). The third cleaning procedure consisted of water spraying for 20 s, coating the bonding surface with Ivoclean (Ivoclar Vivadent, Schaan, Liechtenstein), dispensing Ivoclean with a microbrush for 20 s, water spraying for 20 s and ultrasonic bath with 99% isopropanol (c). The batch numbers of the used materials are given in Table 1.

### 3.3. XPS Analysis

All specimens were examined with X-ray photoelectron spectroscopy (XPS) to quantify the contamination on the surface of the zirconia specimens. For the measurements an Omnicron Full Lab (Omnicron NanoTechnology GmbH, Taunusstein, Germany) equipped with an Al/Mg Kα X-ray source and a VSW 100 hemispherical analyzer was used. All measurements were performed with an excitation energy of 1486.6 eV and in the constant analyzer energy (CAE) mode. A survey spectrum with a CAE of 100 eV was measured to monitor the characteristic lines of the elements present Afterwards three detailed measurements with a CAE of 20 eV were recorded for the peaks of carbon (C1s), silicon (Si2p) and zirconium (Zr3d). The areas under the peak curves were calculated with the EIS software (Omnicron NanoTechnology GmbH, Taunusstein, Germany). The oxygen O1s line dominating the spectrum was not used for the analysis because both the zirconia substrate as well as the residuals will contribute to the line intensity.

The collected data was checked for normal distribution and analyzed using two-way analysis of variance (ANOVA) with a post-hoc Tukey’s HSD test (SPSS v20, Chicago, IL, USA) at a significance level of *p* ≤ 0.05.

## 4. Conclusions

Taking the results and conditions of this study into account, the following conclusions can be drawn:

1. There is no difference between the two investigated silicone disclosing agents concerning the cleanability, with respect to the investigated cleaning processes.
2. Additional ultrasonic cleaning in 99% isopropanol will lead to a significant reduction of contamination.
3. Alternative cleaning processes need to be developed in order to achieve a better removal of contaminants in order to achieve optimal bonding conditions.
